# Patterns and Characteristics of Nicotine Dependence Among Adults With Cigarette Use in the US, 2006-2019

**DOI:** 10.1001/jamanetworkopen.2023.19602

**Published:** 2023-06-23

**Authors:** Beth Han, Emily B. Einstein, Wilson M. Compton

**Affiliations:** 1 National Institute on Drug Abuse, National Institutes of Health, Bethesda, Maryland

## Abstract

**Importance:**

Nicotine dependence increases the risk of persistent smoking, which is the leading preventable cause of morbidity and death. However, evidence regarding the associations of nicotine dependence with age, psychiatric conditions, and sociodemographic characteristics is limited.

**Objective:**

To assess whether and how nicotine dependence among US adults with cigarette use varies by year, age, psychiatric comorbidities, and sociodemographic characteristics.

**Design, Setting, and Participants:**

This exploratory serial cross-sectional study used data from 152 354 US community-dwelling individuals 18 years or older who participated in the 2006-2019 National Surveys on Drug Use and Health. Data analyses were conducted from January 15 to February 15, 2023.

**Exposure:**

Past-month cigarette use. Past-year major depressive episode (MDE) and/or substance use disorder (SUD) based on criteria from the *Diagnostic and Statistical Manual of Mental Disorders* (Fourth Edition), Text Revision.

**Main Outcomes and Measures:**

Past-month nicotine dependence based on criteria from the Nicotine Dependence Syndrome Scale or the Fagerström Test of Nicotine Dependence.

**Results:**

Among 152 354 adults with past-month cigarette use (54.1% male; 40.2% aged 18-34 years; 29.0% aged 35-49 years; 69.8% non-Hispanic White), the adjusted prevalence of nicotine dependence decreased from 59.52% (95% CI, 57.93%-61.10%) in 2006 to 56.00% (95% CI, 54.38%-57.60%) in 2019 (average annual percentage change [AAPC], −0.4%; 95% CI, −0.5% to −0.4%; *P* < .001) and among each examined age group, except for stability among those aged 18 to 25 years (AAPC, −0.5%; 95% CI, −1.4% to 0.4%; *P* = .27). Compared with those 50 years and older with past-month cigarette smoking, the adjusted prevalence of nicotine dependence among those aged 18 to 49 years was 32% lower for those aged 18 to 25 years (adjusted risk ratio [ARR], 0.68; 95% CI, 0.66-0.70), 18% lower for those aged 26 to 34 years (ARR, 0.82; 95% CI, 0.80-0.84), and 6% lower for those aged 35 to 49 years (ARR, 0.94; 95% CI, 0.92-0.96). The adjusted prevalence of nicotine dependence varied by age, MDE and/or SUD status, and sociodemographic characteristics. For example, by 2019, prevalence was 41.27% (95% CI, 39.21%-43.37%) among those aged 18 to 25 years and 64.43% (95% CI, 60.98%-67.74%) among those 50 years and older. Differences in nicotine dependence prevalence between those with co-occurring MDE and SUD and those without both conditions were more than 2 times larger for those 50 years and older vs those aged 18 to 49 years (eg, ages ≥50 years vs 18-25 years: 18.69 percentage point difference [83.32% vs 64.63%] vs 7.67 percentage point difference [48.88% vs 41.21%]; *P* < .001).

**Conclusions and Relevance:**

In this cross-sectional study, there were significant reductions in nicotine dependence prevalence from 2006 to 2019 among US adults with cigarette use and all examined subgroups 26 years and older. Adults 50 years and older (especially those with MDE and/or SUD) had the highest nicotine dependence prevalence compared with other age groups, highlighting the importance of assisting with smoking cessation efforts and addressing nicotine dependence for this older population. Evidence-based tobacco cessation strategies tailored to age and comorbidities are needed.

## Key Points

**Question:**

Does the national prevalence of nicotine dependence vary by year, age, psychiatric comorbidities (substance use disorder and/or depression), and sociodemographic characteristics?

**Findings:**

In this cross-sectional study of 152 354 US community-dwelling adults with past-month cigarette use, the adjusted prevalence of nicotine dependence decreased from 59.52% in 2006 to 56.00% in 2019 among adults with past-month cigarette smoking and among each age group, except for ages 18 to 25 years. Adults 50 years and older (especially those with substance use disorder and/or depression) had the highest prevalence of nicotine dependence.

**Meaning:**

These findings suggest that evidence-based tobacco cessation strategies tailored to age and psychiatric comorbidities are needed.

## Teaser

This cross-sectional study uses data from the National Survey on Drug Use and Health to assess patterns in nicotine dependence prevalence by year, age, psychiatric comorbidities, and sociodemographic characteristics from 2006 to 2019 among US adults with cigarette use.

## Introduction

Nicotine dependence increases the risk of smoking persistence and is the leading preventable cause of morbidity and death.^1^ The prevalence of cigarette smoking has declined in the US over the past decades among adults with and without psychiatric conditions (eg, major depressive episode [MDE] and substance use disorder [SUD]).^2,3^ A nicotine hardening hypothesis has emerged. This hypothesis proposes that declines in nicotine use resulting from population-level tobacco control measures leave a higher proportion of people with intractable nicotine dependence and consumption over time.^4^

However, findings on the hardening hypothesis from existing literature^4,5,6^ are inconsistent. Based on nationally representative data collected from 2001 to 2002 and 2012 to 2013, Grant et al^4^ reported increases in nicotine dependence prevalence among US adults with nicotine use, supporting the hardening hypothesis. Methodological differences between the 2001 to 2002 and 2012 to 2013 data collections may limit comparability for the 2 time periods. Another study^5^ based on representative data from 18 European countries provided empirical evidence against this theory. One study^6^ reviewed 26 population-based studies and did not find that conversion from current to former smoking, number of quit attempts, or success on a given quit attempt decreased over time, suggesting hardening may not be occurring in noninstitutionalized civilian populations with cigarette use.

Moreover, evidence regarding whether and how nicotine dependence varies by age is inconsistent. Studies have reported that a significant decrease in nicotinic activation in the hippocampus (a brain region involved in addiction) occurs around age 40 years,^7,8^ and reduced nicotinic activation is associated with reduced addictive potential of nicotinic agonists.^9^ Another study^10^ based on nationally representative data from 1991 to 1993 reported that US adults 50 years and older who smoke cigarettes had the highest consumption yet experienced the lowest prevalence of nicotine dependence. One study^11^ suggested that this older age group may be more responsive to nonpharmacological cessation programs because of their reduced nicotine dependence. However, the study from 18 European countries^5^ found that high nicotine dependence was associated with older age. Another recent study^12^ based on nationally representative data from China found that the prevalence of nicotine dependence among adults with cigarette use increased with age for men but peaked at age 40 years for women. In addition, although nicotine dependence is associated with lower socioeconomic status^1^ and psychiatric disorders,^13^ little is known about whether and how associations of sociodemographic characteristics and psychiatric conditions with nicotine dependence vary by age.

Given continuing declines in smoking and because the hardening hypothesis could play a role in US tobacco cessation strategies, this study assessed patterns in nicotine dependence among US adults with cigarette use based on nationally representative data from 2006 through 2019. Among adults with cigarette use, patterns and characteristics of nicotine dependence were examined to understand whether and how associations of sociodemographic characteristics and psychiatric conditions with nicotine dependence varied by age.

## Methods

### Data Sources

Data were from 152 354 US community-dwelling individuals 18 years and older who participated in the 2006-2019 National Surveys on Drug Use and Health (NSDUH); the NSDUH provides nationally representative data on cigarette smoking, nicotine dependence, MDE, and SUD among US noninstitutionalized civilian adult populations.^14,15^ The NSDUH data collection protocol was approved by the institutional review board of Research Triangle Institute International. Each participant provided verbal informed consent.^14,15^ This cross-sectional study used publicly available deidentified data from the NSDUH and was exempt from review and the requirement for informed consent per the institutional review board of the National Institutes of Health. The study followed the Strengthening the Reporting of Observational Studies in Epidemiology (STROBE) reporting guideline.

For the NSDUH, data were collected by interviewers through personal visits to households and noninstitutional group facilities. Audio computer-assisted self-administered interviewing was used, providing respondents with a private and confidential way to record answers. From 2006 to 2019, the annual mean weighted screening response rate was 82.9%, and the annual mean weighted interview response rate was 71.4%. Data collected for this cross-sectional study were analyzed from January 15 to February 15, 2023.

### Measures

Among NSDUH respondents who reported past-month cigarette smoking, past-month nicotine dependence was defined as meeting criteria from either the Nicotine Dependence Syndrome Scale or the Fagerström Test of Nicotine Dependence.^13^ Using diagnostic criteria specified in the *Diagnostic and Statistical Manual of Mental Disorders* (Fourth Edition), Text Revision (*DSM-IV-TR*),^2,14,15,16,17^ the NSDUH estimated the prevalence of past year–specific SUD, and respondents were classified as having past-year SUD if they had any of 4 specific SUDs (eg, alcohol use disorder, cannabis use disorder, cocaine use disorder, and heroin use disorder).^2^ The NSDUH also assessed past-year MDE using *DSM-IV-TR* diagnostic criteria.^2,14,15,16,17^ These measures of SUDs and MDE have been found to have good validity and reliability.^18,19^ In addition, the NSDUH asked respondents about sociodemographic characteristics (eg, age, sex, race and ethnicity [including Hispanic; non-Hispanic Black; non-Hispanic White; and other non-Hispance race, including American Indian or Alaska Native, Asian, Native Hawaiian or other Pacific Islander, and more than 1 race]), educational attainment, employment status, family income, marital status, health insurance status, and metropolitan statistical area [MSA] status). Self-reported race and ethnicity classification was included because tobacco use has previously been documented to vary according to these socially determined factors.^1^ Additional details about NSDUH methods and survey questionnaires have been published previously.^14,15,16^

### Statistical Analysis

Bivariable and multivariable logistic regression analyses were conducted to understand factors associated with past-month nicotine dependence among adults with past-month cigarette smoking. Model-adjusted prevalence (predicted marginal proportion) and differences in model-adjusted prevalence (differences in predicted marginal proportions) were estimated. Multicollinearity and potential interaction effects were examined. Although multicollinearity was not found in the final pooled model, significant interaction effects were identified between age and selected covariates. Thus, stratified multivariable logistic regression models by age were conducted.

All of these analyses used SUDAAN software, release 11.0.3 (RTI International),^20^ to account for the complex sample design and sample weights used in the NSDUH. The Joinpoint regression program, version 4.8.0.1 (National Cancer Institute),^21^ was used to test for significant changes in nonlinear patterns in the adjusted prevalence of nicotine dependence using permutation tests and to estimate average annual percentage changes (AAPCs) from 2006 to 2019, which are considered valid even if the Joinpoint models suggest changes in patterns during a given study period.^22^ For each analysis, 2-tailed *P* < .05 was considered statistically significant.

## Results

### Overall Patterns and Characteristics of Nicotine Dependence

Among 152 354 community-dwelling adults with past-month cigarette use, 54.1% were male, 45.9% were female, 18.8% were ages 18 to 25 years, 21.4% were ages 26 to 34 years, 29.0% were ages 35 to 49 years, and 30.8% were 50 years or older. With regard to race and ethnicity, 12.2% were Hispanic, 12.3% were non-Hispanic Black, 69.8% were non-Hispanic White, and 5.6% were of other non-Hispanic race (including American Indian or Alaska Native, Asian, Native Hawaiian or other Pacific Islander, and more than 1 race). Overall, the adjusted prevalence of nicotine dependence decreased from 59.52% (95% CI, 57.93%-61.10%) in 2006 to 56.00% (95% CI, 54.38%-57.60%) in 2019 (AAPC, −0.4%; 95% CI,−0.5% to −0.4%; *P* < .001), representing a percentage point difference of 3.52 and a percentage change of 5.91% (Table 1). Prevalence was lower from 2012 to 2019 (eg, 2012: 56.83% [95% CI, 55.30%-58.34%]; 2015: 56.80% [95% CI, 55.46%-58.13%]; 2018: 55.64% [95% CI, 54.39%-56.87%]) than in 2006. Moreover, higher prevalence of nicotine dependence was associated with older age. Compared with those 50 years and older with past-month cigarette smoking, the adjusted prevalence of nicotine dependence was 32% lower among those aged 18 to 25 years (adjusted risk ratio [ARR], 0.68; 95% CI, 0.66-0.70), 18% lower among those aged 26 to 34 years (ARR, 0.82; 95% CI, 0.80-0.84), and 6% lower among those aged 35 to 49 years (ARR, 0.94; 95% CI, 0.92-0.96) (Table 2).

**Table 1.** Adjusted Past-Month Prevalence of Nicotine Dependence Among Adults With Past-Month Cigarette Smoking in the US^a^

Characteristic	Adjusted prevalence (95% CI)				
	Pooled model (N = 152 354)^b^^,^^c^	Age group, y			
		18-25 (n = 71 865)	26-34 (n = 29 895)	35-49 (n = 33 497)	≥50 (n = 17 097)
Survey year					
2006^d^	59.52 (57.93-61.10)	43.49 (41.94-45.05)	53.04 (49.85-56.21)	65.45 (62.94-67.87)	68.02 (64.12-71.68)
2007	57.93 (56.63-59.22)	43.83 (42.63-45.05)	53.16 (50.42-55.88)	62.61 (59.93-65.22)	65.16 (61.34-68.80)
2008	59.40 (58.06-60.72)	44.16 (42.67-45.67)	54.93 (52.56-57.29)	63.85 (61.03-66.57)	67.49 (64.01-70.78)
2009	57.61 (56.11-59.10)	41.98 (40.53-43.46)	50.17 (47.34-52.99)	62.97 (60.67-65.22)	67.37 (63.14-71.34)
2010	57.62 (56.06-59.16)	41.32 (39.81-42.85)	52.83 (50.50-55.14)	61.94 (59.41-64.40)^e^	66.88 (62.77-70.74)
2011	58.11 (56.50-59.69)	42.20 (40.50-43.91)	50.60 (47.75-53.45)	63.52 (60.66-66.29)	68.07 (64.26-71.65)
2012	56.83 (55.30-58.34)^e^	42.96 (41.40-44.55)	49.34 (45.90-52.79)	60.93 (58.30-63.51)^e^	66.64 (62.99-70.09)
2013	56.78 (55.31-58.23)^e^	42.48 (40.72-44.26)	48.54 (46.13-50.95)^e^	60.00 (56.58-63.32)^e^	68.27 (64.68-71.65)
2014	57.05 (55.72-58.38)^e^	44.04 (41.59-46.51)	52.39 (50.21-54.57)	58.83 (56.54-61.08)^e^	66.55 (64.19-68.83)
2015	56.80 (55.46-58.13)^e^	44.08 (41.69-46.49)	51.23 (48.94-53.52)	59.13 (57.59-60.66)^e^	66.18 (63.08-69.14)
2016	56.48 (55.32-57.64)^e^	42.65 (40.52-44.80)	50.51 (48.48-52.54)	58.14 (55.63-60.61)^e^	67.25 (64.69-69.71)
2017	56.40 (54.90-57.88)^e^	43.84 (41.58-46.13)	48.93 (46.73-51.14)^e^	58.58 (56.10-61.01)^e^	67.21 (64.14-70.13)
2018	55.64 (54.39-56.87)^e^	42.02 (39.83-44.24)	51.54 (48.70-54.36)	59.72 (57.29-62.10)^e^	63.27 (60.84-65.63)^e^
2019	56.00 (54.38-57.60)^e^	41.27 (39.21-43.37)	50.64 (47.99-53.30)	60.71 (58.27-63.09)^e^	64.43 (60.98-67.74)
Age group, y					
18-25	45.48 (44.48-46.49)^e^	NA	NA	NA	NA
26-34	54.94 (54.19-55.69)^e^	NA	NA	NA	NA
35-49	62.97 (62.19-63.76)^e^	NA	NA	NA	NA
≥50^d^	66.85 (65.45-68.22)	NA	NA	NA	NA
Sex					
Men	57.96 (57.46-58.46)^e^	42.96 (42.25-43.68)	52.11 (51.14-53.08)^e^	62.13 (61.19-63.07)^e^	67.28 (66.11-68.43)
Women^d^	56.68 (56.01-57.34)	42.89 (42.09-43.68)	50.22 (49.14-51.31)	60.53 (59.52-61.54)	65.91 (64.45-67.34)
Race and ethnicity					
Hispanic	35.69 (34.17-37.25)^e^	23.38 (22.14-24.45)^e^	28.48 (26.84-30.19)^e^	37.37 (35.26-39.52)^e^	46.69 (42.76-50.67)^e^
Non-Hispanic Black	51.76 (50.50-53.01)^e^	40.74 (39.46-42.04)^e^	50.39 (48.41-52.38)^e^	54.93 (52.73-57.11)^e^	56.37 (53.50-59.20)^e^
Non-Hispanic White^d^	62.67 (62.22-63.11)	48.29 (47.62-48.97)	57.62 (56.78-58.45)	67.40 (66.58-68.21)	70.50 (69.57-71.41)
Non-Hispanic other race^f^	53.15 (51.21-55.08)^e^	42.10 (39.80-44.43)^e^	44.30 (41.65-46.98)^e^	55.49 (52.27-58.66)^e^	63.79 (59.36-67.99)^e^
Employment status					
Full time^d^	56.64 (55.97-57.31)	44.31 (43.54-45.08)	49.09 (48.10-50.08)	60.14 (59.26-61.02)	65.72 (62.86-68.47)
Part time	55.21 (54.06-56.35)^e^	36.22 (35.18-37.28)^e^	50.28 (48.05-52.50)	60.23 (58.14-62.28)	67.75 (62.71-72.41)
Unemployed	60.50 (58.80-62.17)^e^	48.47 (47.38-49.56)^e^	57.38 (55.06-59.67)^e^	62.85 (60.49-65.16)^e^	67.26 (65.78-68.70)
Other	60.33 (59.48-61.17)^e^	43.35 (42.12-44.60)	57.77 (55.72-59.78)^e^	66.00 (64.13-67.82)^e^	66.01 (64.33-67.66)
Family income, $					
<20 000	59.65 (58.73-60.57)^e^	42.91 (42.07-43.76)^e^	53.56 (51.98-55.14)^e^	65.10 (63.39-66.77)^e^	68.91 (66.91-70.83)^e^
20 000-49 999	58.75 (58.17-59.34)^e^	44.20 (43.35-45.04)^e^	51.83 (50.64-53.02)^e^	63.60 (62.52-64.66)^e^	67.84 (66.41-69.23)^e^
50 000-74 999	56.70 (55.55-57.84)^e^	43.08 (41.48-44.70)^e^	50.50 (48.61-52.38)^e^	60.68 (58.91-62.43)^e^	65.53 (62.73-68.22)
≥75 000^d^	53.63 (52.66-54.61)	40.55 (39.46-41.65)	48.37 (46.93-49.82)	56.30 (54.60-57.98)	62.88 (60.26-65.42)
Educational attainment					
Less than high school	66.70 (65.79-67.59)^e^	54.79 (53.66-55.91)^e^	63.71 (61.71-65.66)^e^	69.24 (67.66-70.79)^e^	73.68 (71.64-75.62)^e^
High school diploma	61.52 (60.81-62.23)^e^	47.60 (46.74-48.46)^e^	57.38 (56.09-58.66)^e^	65.42 (64.29-66.53)^e^	69.13 (67.40-70.80)^e^
Some college	54.60 (53.93-55.27)^e^	35.08 (34.22-35.96)^e^	49.92 (48.79-51.06)^e^	59.78 (58.42-61.13)^e^	64.89 (63.22-66.53)^e^
College degree^d^	41.53 (40.32-42.75)	23.21 (21.65-24.84)	32.16 (30.42-33.94)	46.86 (44.98-48.76)	54.28 (51.22-57.31)
Health insurance status					
Medicaid	62.78 (61.50-64.05)^e^	52.83 (51.45-54.20)^e^	58.36 (56.53-60.15)^e^	66.44 (64.32-68.50)^e^	68.52 (65.12-71.73)
Private only^d^	54.55 (53.92-55.18)	36.55 (35.85-37.25)	47.66 (46.40-48.91)	59.00 (58.02-59.98)	66.15 (64.46-67.79)
Uninsured	59.34 (58.29-60.38)^e^	48.85 (47.84-49.87)^e^	53.02 (51.56-54.46)^e^	63.50 (61.85-65.12)^e^	66.24 (63.48-68.89)
Other	57.88 (56.94-58.82)^e^	41.37 (40.08-42.68)^e^	51.67 (49.18-54.16)^e^	63.51 (61.47-65.50)^e^	66.85 (65.23-68.42)
Marital status					
Married^d^	57.01 (56.31-57.71)	46.38 (45.00-47.77)	49.36 (48.22-50.50)	59.87 (58.87-60.86)	66.04 (64.46-67.60)
Divorced or separated	61.03 (60.15-61.90)^e^	52.57 (49.55-55.57)^e^	56.47 (54.55-58.36)^e^	63.39 (62.14-64.63)^e^	67.08 (65.51-68.61)
Never married	57.90 (56.80-58.99)	42.24 (41.64-42.83)^e^	51.11 (50.14-52.07)^e^	62.45 (60.89-63.99)^e^	67.98 (64.99-70.83)
Other	64.26 (61.38-67.05)^e^	58.81 (50.08-67.01)^e^	69.19 (60.11-76.99)^e^	60.94 (55.93-65.73)	66.58 (63.88-69.18)
Metropolitan statistical area					
Large metropolitan	55.99 (55.39-56.59)^e^	42.24 (41.41-43.07)^e^	49.06 (48.03-50.09)^e^	59.70 (58.72-60.67)^e^	65.71 (64.29-67.10)^e^
Small metropolitan	58.14 (57.50-58.77)^e^	43.38 (42.62-44.14)	52.98 (51.64-54.31)^e^	61.96 (60.73-63.18)^e^	67.12 (65.47-68.74)
Nonmetropolitan^d^	59.90 (59.10-60.71)	43.95 (42.77-45.14)	55.17 (53.52-56.82)	65.12 (63.66-66.54)	68.00 (66.21-69.74)
Psychiatric condition					
MDE and SUD	66.96 (64.82-69.03)^e^	48.88 (46.87-50.88)^e^	57.76 (54.62-60.83)^e^	67.86 (63.96-71.52)	83.32 (78.16-87.46)^e^
MDE without SUD	65.37 (64.00-66.72)^e^	45.74 (43.76-47.74)	59.28 (56.42-62.08)^e^	70.44 (68.32-72.48)^e^	76.71 (73.92-79.28)
SUD without MDE^d^	61.98 (60.79-63.15)	44.99 (44.04-45.94)	53.95 (52.33-55.56)	63.53 (61.34-65.67)	76.42 (73.34-79.25)
Neither MDE nor SUD	55.62 (55.12-56.12)^e^	41.21 (40.54-41.88)^e^	49.48 (48.65-50.31)^e^	59.90 (59.07-60.72)^e^	64.63 (63.51-65.74)^e^

Abbreviations: MDE, major depressive episode; NA, not applicable; SUD, substance use disorder (includes alcohol use disorder, cannabis use disorder, cocaine use disorder, and heroin use disorder).

^a^ Results of the final pooled and stratified multivariable logistic regression models with these variables. All data were obtained from the 2006-2019 National Surveys on Drug Use and Health.

^b^ The final sample size was 152 354 rather than 154 394 due to the unknown MDE status of 2040 respondents (1.3%).

^c^ Also includes the following interaction effects: race and ethnicity and age (*P* < .001), employment status and age (*P* < .001), family income and age (*P* = .007), educational attainment and age (*P* < .001), health insurance status and age (*P* < .001), marital status and age (*P* < .001), metropolitan statistical area and age (*P* = .01), and psychiatric condition and age (*P* < .001).

^d^ Reference group.

^e^ Estimates were significantly different (*P* < .05) from the corresponding reference group.

^f^ Includes non-Hispanic American Indian or Alaska Native, non-Hispanic Asian, non-Hispanic Native Hawaiian or other Pacific Islander, and non-Hispanic more than 1 race. Race and ethnicity were determined according to National Survey on Drug Use and Health respondents’ self-classification of racial and ethnic origin and identification based on US Census Bureau classifications.

**Table 2.** Characteristics Associated With Past-Month Nicotine Dependence Among Adults With Past-Month Cigarette Smoking in the US^a^

Characteristic	Adjusted risk ratio (95% CI)				
	Pooled model (N = 152 354)^b^^,^^c^	Age group, y			
		18-25 (n = 71 865)	26-34 (n = 29 895)	35-49 (n = 33 497)	≥50 (n = 17 097)
Survey year					
2006	1 [Reference]	1 [Reference]	1 [Reference]	1 [Reference]	1 [Reference]
2007	0.97 (0.94-1.01)	1.01 (0.97-1.05)	1.00 (0.93-1.08)	0.96 (0.90-1.01)	0.96 (0.88-1.04)
2008	1.00 (0.96-1.03)	1.02 (0.97-1.07)	1.04 (0.97-1.11)	0.98 (0.92-1.04)	0.99 (0.92-1.07)
2009	0.97 (0.94-1.00)	0.97 (0.92-1.02)	0.95 (0.87-1.03)	0.96 (0.91-1.01)	0.99 (0.92-1.07)
2010	0.97 (0.93-1.01)	0.95 (0.90-1.00)	1.00 (0.92-1.08)	0.95 (0.90-0.99)^d^	0.98 (0.91-1.07)
2011	0.98 (0.94-1.02)	0.97 (0.93-1.02)	0.95 (0.88-1.04)	0.97 (0.91-1.03)	1.00 (0.93-1.08)
2012	0.96 (0.92-0.99)^d^	0.99 (0.94-1.04)	0.93 (0.85-1.01)	0.93 (0.88-0.99)^d^	0.98 (0.91-1.05)
2013	0.95 (0.92-0.99)^d^	0.98 (0.93-1.03)	0.92 (0.84-0.99)^d^	0.92 (0.86-0.98)^d^	1.00 (0.93-1.08)
2014	0.96 (0.93-0.99)^d^	1.01 (0.95-1.08)	0.99 (0.92-1.06)	0.90 (0.85-0.95)^d^	0.98 (0.92-1.04)
2015	0.95 (0.92-0.99)^d^	1.01 (0.95-1.08)	0.97 (0.90-1.04)	0.90 (0.86-0.95)^d^	0.97 (0.91-1.05)
2016	0.95 (0.92-0.98)^d^	0.98 (0.92-1.04)	0.95 (0.89-1.02)	0.89 (0.84-0.94)^d^	0.99 (0.93-1.06)
2017	0.95 (0.91-0.98)^d^	1.01 (0.95-1.07)	0.92 (0.86-0.99)^d^	0.90 (0.85-0.95)^d^	0.99 (0.92-1.06)
2018	0.94 (0.90-0.97)^d^	0.97 (0.91-1.03)	0.97 (0.90-1.05)	0.91 (0.86-0.97)^d^	0.93 (0.87-0.99)^d^
2019	0.94 (0.90-0.98)^d^	0.95 (0.89-1.01)	0.96 (0.88-1.04)	0.93 (0.88-0.98)^d^	0.95 (0.88-1.02)
Age group, y					
18-25	0.68 (0.66-0.70)^d^	NA	NA	NA	NA
26-34	0.82 (0.80-0.84)^d^	NA	NA	NA	NA
35-49	0.94 (0.92-0.96)^d^	NA	NA	NA	NA
≥50	1 [Reference]	NA	NA	NA	NA
Sex					
Men	1.02 (1.01-1.04)^d^	1.00 (0.98-1.03)	1.04 (1.01-1.07)^d^	1.03 (1.00-1.05)^d^	1.02 (0.99-1.05)
Women	1 [Reference]	1 [Reference]	1 [Reference]	1 [Reference]	1 [Reference]
Race and ethnicity					
Hispanic	0.57 (0.55-0.60)^d^	0.48 (0.46-0.51)^d^	0.49 (0.47-0.53)^d^	0.55 (0.52-0.59)^d^	0.62 (0.61-0.72)^d^
Non-Hispanic Black	0.83 (0.81-0.85)^d^	0.84 (0.82-0.87)^d^	0.88 (0.84-0.91)^d^	0.82 (0.73-0.85)^d^	0.80 (0.76-0.84)^d^
Non-Hispanic White	1 [Reference]	1 [Reference]	1 [Reference]	1 [Reference]	1 [Reference]
Non-Hispanic other race^e^	0.85 (0.82-0.88)^d^	0.87 (0.83-0.92)^d^	0.76 (0.72-0.82)^d^	0.82 (0.78-0.87)^d^	0.91 (0.85-0.97)^d^
Employment status					
Full time	1 [Reference]	1 [Reference]	1 [Reference]	1 [Reference]	1 [Reference]
Part time	0.98 (0.95-0.99)^d^	0.82 (0.79-0.84)^d^	1.02 (0.76-1.08)	1.00 (0.97-1.04)	1.00 (0.95-1.04)
Unemployed	1.07 (1.04-1.10)^d^	1.09 (1.06-1.13)^d^	1.17 (1.11-1.23)^d^	1.05 (1.00-1.09)^d^	1.03 (0.95-1.11)
Other	1.06 (1.05-1.09)^d^	0.98 (0.95-1.01)	1.18 (1.13-1.23)^d^	1.10 (1.06-1.13)^d^	1.02 (0.98-1.06)
Family income, $					
<20 000	1.11 (1.08-1.14)^d^	1.06 (1.02-1.09)^d^	1.11 (1.06-1.16)^d^	1.16 (1.10-1.21)^d^	1.10 (1.04-1.16)^d^
20 000-49 999	1.10 (1.07-1.12)^d^	1.09 (1.06-1.12)^d^	1.08 (1.03-1.11)^d^	1.13 (1.09-1.17)^d^	1.08 (1.03-1.13)^d^
50 000-74 999	1.06 (1.03-1.08)^d^	1.06 (1.02-1.11)^d^	1.04 (1.00-1.09)^d^	1.08 (1.04-1.12)^d^	1.04 (0.98-1.11)
≥75 000	1 [Reference]	1 [Reference]	1 [Reference]	1 [Reference]	1 [Reference]
Educational attainment					
Less than high school	1.61 (1.55-1.66)^d^	2.36 (2.20-2.53)^d^	1.98 (1.86-2.13)^d^	1.48 (1.41-1.55)^d^	1.36 (1.27-1.45)^d^
High school diploma	1.48 (1.44-1.53 ^d^	2.05 (1.91-2.20)^d^	1.78 (1.68-1.90)^d^	1.40 (1.34-1.46)^d^	1.27 (1.20-1.35)^d^
Some college	1.32 (1.27-1.36)^d^	1.51 (1.41-1.62)^d^	1.55 (1.46-1.65)^d^	1.28 (1.22-1.34)^d^	1.20 (1.12-1.27)^d^
College degree	1 [Reference]	1 [Reference]	1 [Reference]	1 [Reference]	1 [Reference]
Health insurance status					
Medicaid	1.15 (1.12-1.18)^d^	1.45 (1.40-1.49)^d^	1.23 (1.18-1.28)^d^	1.13 (1.09-1.17)^d^	1.04 (0.98-1.10)
Private only	1 [Reference]	1 [Reference]	1 [Reference]	1 [Reference]	1 [Reference]
Uninsured	1.09 (1.07-1.11)^d^	1.34 (1.30-1.37)^d^	1.11 (1.07-1.16)^d^	1.08 (1.04-1.11)^d^	1.00 (0.96-1.05)
Other	1.06 (1.04-1.08)^d^	1.13 (1.09-1.18)^d^	1.08 (1.02-1.15)^d^	1.08 (1.04-1.11)^d^	1.01 (0.97-1.05)
Marital status					
Married	1 [Reference]	1 [Reference]	1 [Reference]	1 [Reference]	1 [Reference]
Divorced or separated	1.07 (1.05-1.09)^d^	1.13 (1.06-1.21)^d^	1.14 (1.10-1.19)^d^	1.06 (1.03-1.09)^d^	1.02 (0.98-1.05)
Never married	1.02 (0.99-1.04)	0.91 (0.88-0.94)^d^	1.04 (1.01-1.07)^d^	1.04 (1.01-1.08)^d^	1.03 (0.98-1.08)
Other	1.13 (1.08-1.18)^d^	1.27 (1.09-1.47)^d^	1.40 (1.24-1.59)^d^	1.02 (0.94-1.11)	1.01 (0.96-1.06)
Metropolitan statistical area					
Large metropolitan	0.94 (0.92-0.95)^d^	0.96 (0.93-0.99)^d^	0.89 (0.86-0.92)^d^	0.92 (0.89-0.94)^d^	0.97 (0.94-0.99)^d^
Small metropolitan	0.97 (0.96-0.99)^d^	0.99 (0.96-1.02)	0.96 (0.93-0.99)^d^	0.95 (0.92-0.98)^d^	0.99 (0.96-1.02)
Nonmetropolitan	1 [Reference]	1 [Reference]	1 [Reference]	1 [Reference]	1 [Reference]
Psychiatric condition					
MDE and SUD	1.08 (1.04-1.12)^d^	1.09 (1.04-1.14)^d^	1.07 (1.01-1.14)^d^	1.07 (1.00-1.14)	1.09 (1.02-1.17)^d^
MDE without SUD	1.06 (1.03-1.08)^d^	1.02 (0.97-1.07)	1.10 (1.04-1.16)^d^	1.11 (1.06-1.16)^d^	1.00 (0.96-1.05)
SUD without MDE	1 [Reference]	1 [Reference]	1 [Reference]	1 [Reference]	1 [Reference]
Neither MDE nor SUD	0.90 (0.88-0.92)^d^	0.92 (0.89-0.94)^d^	0.92 (0.89-0.95)^d^	0.94 (0.91-0.98)^d^	0.85 (0.81-0.88)^d^

Abbreviations: MDE, major depressive episode; NA, not applicable; SUD, substance use disorder (includes alcohol use disorder, cannabis use disorder, cocaine use disorder, and heroin use disorder).

^a^ Results of the final pooled and stratified multivariable logistic regression models with these variables. All data were obtained from the 2006 to 2019 National Surveys on Drug Use and Health.

^b^ The final sample size was 152 354 rather than 154 394 due to the unknown MDE status of 2040 respondents (1.3%).

^c^ Also includes the following interaction effects: race and ethnicity and age (*P* < .001), employment status and age (*P* < .001), family income and age (*P* = .007), educational attainment and age (*P* < .001), health insurance status and age (*P* < .001), marital status and age (*P* < .001), metropolitan statistical area and age (*P* = .01), and psychiatric condition and age (*P* < .001).

^d^ Estimates were significantly different (*P* < .05) from the corresponding reference group.

^e^ Includes non-Hispanic American Indian or Alaska Native, non-Hispanic Asian, non-Hispanic Native Hawaiian or other Pacific Islander, and non-Hispanic more than 1 race. Race and ethnicity were determined according to National Survey on Drug Use and Health respondents’ self-classification of racial and ethnic origin and identification based on US Census Bureau classifications.

Nicotine dependence was also positively associated with male sex, non-Hispanic White race and ethnicity, non–full-time employment status, low family income, low educational attainment, nonprivate health insurance status, divorced or separated status, residence in a non-MSA, and the presence of MDE and/or SUD (Table 1 and Table 2). The pooled model identified 8 interaction effects (race and ethnicity and age, employment status and age, family income and age, educational attainment and age, health insurance status and age, marital status and age, MSA and age, and psychiatric condition and age), suggesting that associations of these characteristics with nicotine dependence varied by age. Thus, age-stratified multivariable logistic regression analysis was conducted.

### Patterns in Nicotine Dependence Prevalence by Age

**Figure 1.**
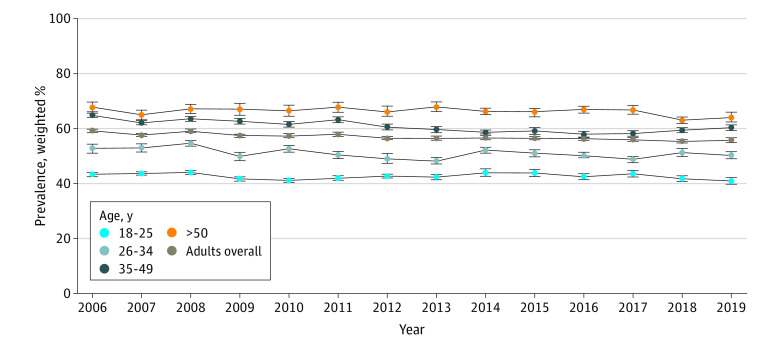
Patterns in Adjusted Past-Month Prevalence of Nicotine Dependence Among US Adults With Past-Month Cigarette Smoking by Age Data were obtained from the 2006-2019 National Surveys on Drug Use and Health. Each estimate was adjusted for sex, race and ethnicity, educational attainment, family income, employment status, health insurance status, marital status, metropolitan statistical area status, major depressive episode, and substance use disorder (includes alcohol use disorder, cannabis use disorder, cocaine use disorder, and heroin use disorder). Error bars represent SEs.

Among adults with past-month cigarette smoking from 2006 to 2019, the adjusted prevalence of nicotine dependence remained statistically unchanged among those aged 18 to 25 years (AAPC, −0.5%; 95% CI, −1.4% to 0.4%; *P* = .27) (Figure 1; Table 1 and Table 2) but decreased among those aged 26 to 49 years (ages 26-34 years: AAPC, −0.5% [95% CI, −0.9% to −0.2%]; *P* = .008; ages 35-49 years: AAPC, −0.8% [95% CI, −1.0% to −0.5]; *P* < .001) and those 50 years and older (AAPC, −0.3%; 95% CI, −0.5% to 0.0%; *P* = .04). By 2019, adjusted nicotine dependence prevalence was 41.27% (95% CI, 39.21%-43.37%) among those aged 18 to 25 years, 50.64% (95% CI, 47.99%-53.30%) among those aged 26 to 34 years, 60.71% (95% CI, 58.27%-63.09%) among those aged 34 to 49 years, and 64.43% (95% CI, 60.98%-67.74%) among those 50 years and older.

### Nicotine Dependence Prevalence by Age, Sex, and Race and Ethnicity

Nicotine dependence prevalence was 4% higher in men aged 26 to 34 years (ARR, 1.04; 95% CI, 1.01-1.07) and 3% higher in men aged 35 to 49 years (ARR, 1.03; 95% CI, 1.00-1.05) compared with women (Table 2). Prevalence was lower among minority racial and ethnic adults than non-Hispanic White adults across examined age groups; difference in prevalence ranged from 9% lower for non-Hispanic individuals of other race who were 50 years and older (ARR, 0.91; 95% CI, 0.85-0.97) to 52% lower for Hispanic individuals aged 18 to 25 years (ARR, 0.48; 95% CI, 0.46-0.51). However, compared with those 50 years and older, differences in nicotine dependence prevalence between non-Hispanic White vs non-Hispanic Black groups were smaller for those aged 18 to 25 years (difference, 7.55 percentage points [48.29% vs 40.74%] vs 14.13 percentage points [70.50% vs 56.37%]; *P* < .001) and those aged 26 to 34 years (difference, 7.23 percentage points [57.62% vs 50.39%] vs 14.13 percentage points [70.50% vs 56.37%]; *P* < .001) (Table 1). Compared with those 50 years and older, differences in prevalence between non-Hispanic White and Hispanic groups were larger for those aged 26 to 34 years (difference, 29.14 percentage points [57.62% vs 28.48%] vs 23.81 percentage points [70.50% vs 46.69%]; *P* = .004) and aged 35 to 49 years (difference, 30.03 percentage points [67.40% vs 37.37%] vs 23.81 percentage points [70.50% vs 46.69%]; *P* = .002).

### Nicotine Dependence Prevalence by Age, Family Income, Employment Status, Health Insurance, and Marital Status

Compared with adults with a family income of $75 000 or higher, nicotine dependence prevalence was higher among adults with a family income of less than $50 000 across all age groups, ranging from 6% higher for those aged 18 to 25 years with income of less than $20 000 (ARR, 1.06; 95% CI, 1.02-1.09) to 16% higher for those aged 35 to 49 years with income of less than $20 000 (ARR, 1.16; 95% CI, 1.10-1.21) (Table 2). In addition, prevalence was higher among those aged 18 to 49 years with a family income between $50 000 and $74 999 vs $75 000 or higher, ranging from 4% higher for those aged 26 to 34 years (ARR, 1.04; 95% CI, 1.00-1.09) to 8% higher for those aged 35 to 49 years (ARR, 1.08; 95% CI, 1.04-1.12). Differences in nicotine dependence prevalence between adults with family incomes of less than $20 000 vs $75 000 or higher were greater among those aged 35 to 49 years than those aged 18 to 25 years (difference, 8.80% percentage points [65.10% vs 56.30%] vs 2.36% percentage points [42.91% vs 40.55%]; *P* < .001) (Table 1).

Nicotine dependence prevalence did not differ by employment, health insurance, or marital status among those 50 years and older with cigarette use. However, prevalence was 18% lower among those aged 18 to 25 years with part-time employment than their counterparts with full-time employment (ARR, 0.82; 95% CI, 0.79-0.84) (Table 2). Among adults aged 18 to 49 years, prevalence was higher among those who were unemployed vs those who had full-time employment, ranging from 5% higher for those aged 35 to 49 years (ARR, 1.05; 95% CI, 1.00-1.09) to 17% higher for those aged 26 to 34 years (ARR, 1.17; 95% CI, 1.11-1.23).

Among adults aged 18 to 49 years, nicotine dependence prevalence was higher for those with Medicaid vs private insurance only, ranging from 13% higher among those aged 35 to 49 years (ARR, 1.13; 95% CI, 1.09-1.17) to 45% higher among those aged 18 to 25 years (ARR, 1.45; 95% CI, 1.40-1.49) (Table 2). In addition, prevalence was higher among those aged 18 to 49 years with no insurance vs private insurance only, ranging from 8% higher for those aged 35 to 49 years (ARR, 1.08; 95% CI, 1.04-1.11) to 34% higher for those aged 18 to 25 years (ARR, 1.34; 95% CI, 1.30-1.37). Among adults aged 18 to 49 years, prevalence was higher for those who were divorced or separated vs married, ranging from 6% higher among those aged 35 to 49 years (ARR, 1.06; 95% CI, 1.03-1.09) to 14% higher among those aged 26 to 34 years (ARR, 1.14; 95% CI, 1.10-1.19). Among adults aged 26 to 49 years, prevalence was 4% higher for those who were never married vs married (ages 26-34 years: ARR, 1.04 [95% CI, 1.01-1.07]; ages 35-49 years: ARR, 1.04 [95% CI, 1.01-1.08]). Among adults aged 18 to 25 years, prevalence was 9% lower for those who were never married vs married (ARR, 0.91; 95% CI, 0.88-0.94).

### Nicotine Dependence Prevalence by Age, Educational Attainment, and MSA

Across age groups, nicotine dependence prevalence was higher among those without a college degree vs those with a college degree, ranging from 20% higher for those 50 years and older with some college education (ARR, 1.20; 95% CI, 1.12-1.27) to 98% higher for those aged 26 to 34 years (ARR, 1.98; 95% CI, 1.86-2.13) and 136% higher for those aged 18 to 25 years with less than a high school diploma (ARR, 2.36; 95% CI, 2.20-2.53) (Table 2). Compared with adults 50 years and older, differences in nicotine dependence prevalence between those without a high school diploma vs those with a college degree were higher among those aged 18 to 25 years (difference, 31.58 percentage points [54.79% vs 23.21%] vs 19.40 percentage points [73.68% vs 54.28%]; *P* < .001) and those aged 26 to 34 years (difference, 31.55 percentage points [63.71% vs 32.16%] vs 19.40 percentage points; *P* < .001) (Table 1).

Across age groups, nicotine dependence prevalence was lower among those residing in large MSAs than those in non-MSAs, ranging from 3% lower for those 50 years and older (ARR, 0.97; 95% CI, 0.94-0.99) to 11% lower for those aged 26 to 34 years (ARR, 0.89; 95% CI, 0.86-0.92) (Table 2). Compared with adults 50 years and older, differences in nicotine dependence prevalence between those residing in non-MSAs vs large MSAs were greater among those aged 26 to 34 years (difference, 6.11 percentage points [55.17% vs 49.06%] vs 2.29 percentage points [68.00% vs 65.71%]; *P* = .01) and those aged 35 to 49 years (difference, 5.42 percentage points [65.12% vs 59.70%] vs 2.29 percentage points [68.00% vs 65.71%]; *P* = .03) (Table 1). Prevalence was lower among adults aged 26 to 49 years residing in small MSAs vs non-MSAs, ranging from 4% lower for those aged 26 to 34 years (ARR, 0.96; 95% CI, 0.93-0.99) to 5% lower for those aged 35 to 49 years (ARR, 0.95; 95% CI, 0.92-0.98) (Table 2).

### Nicotine Dependence Prevalence by Age, SUD, and MDE

**Figure 2.**
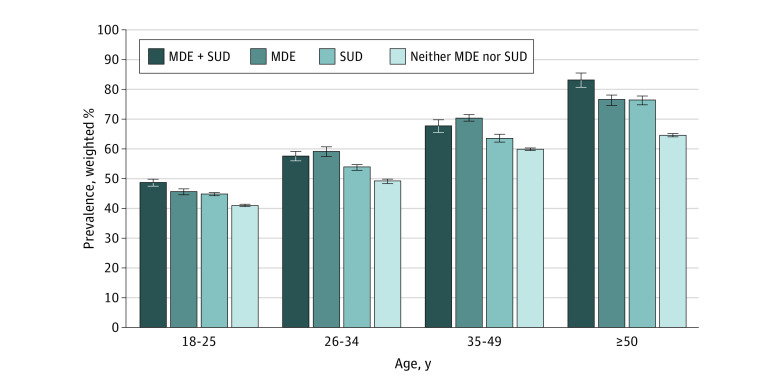
Adjusted Past-Month Prevalence of Nicotine Dependence Among US Adults With Past-Month Cigarette Smoking by Age and Psychiatric Condition Data were obtained from the 2006-2019 National Surveys on Drug Use and Health. Each estimate was adjusted for sex, race and ethnicity, educational attainment, family income, employment status, health insurance status, marital status, metropolitan statistical area status, and survey year. Error bars represent SEs. MDE indicates major depressive episode; and SUD, substance use disorder.

Overall, nicotine dependence was higher among adults with cigarette use who also had SUD and/or MDE than among those without these comorbid conditions, but the pattern of associations varied according to age group (Table 1 and Table 2; Figure 2). For example, the differences in nicotine dependence prevalence between those with co-occurring SUD and MDE and those with neither condition were more than 2 times larger for adults 50 years and older vs those aged 18 to 49 years (eg, ages ≥50 years vs 18-25 years: 18.69 [83.32 vs 64.63] percentage point difference vs 7.67 [48.88 vs 41.21] percentage point difference; *P* < .001; ages ≥50 years vs 35-49 years: 18.69 vs 8.28 percentage point difference [57.76% vs 49.48%]; *P* < .001) (Table 1). In all age groups of adults with cigarette use who had neither SUD nor MDE, nicotine dependence prevalence was lower than among adults with cigarette use who had SUD but not MDE, ranging from 6% lower for those aged 35 to 49 years (ARR, 0.94; 95% CI, 0.91-0.98) to 15% lower for those 50 years and older (ARR, 0.85; 95% CI, 0.81-0.88) (Table 2). However, also compared with those with SUD but without MDE, nicotine dependence prevalence was higher only among those aged 18 to 25 years (ARR, 1.09; 95% CI, 1.04-1.14), those aged 26 to 34 years (ARR, 1.07; 95% CI, 1.01-1.14), and those 50 years and older (ARR, 1.09; 95% CI, 1.02-1.17) with co-occurring SUD and MDE. Prevalence was also higher among those aged 26 to 34 years (ARR, 1.10; 95% CI, 1.04-1.16) and those aged 35 to 49 years (ARR, 1.11; 95% CI, 1.06-1.16) with MDE but without SUD compared with those with SUD but without MDE.

## Discussion

Consistent with the findings based on representative data from 18 European countries,^5^ this cross-sectional study found that the adjusted prevalence of nicotine dependence declined from 2006 to 2019 among the US general adult population who smoked cigarettes and among each examined age group, except for stability among those aged 18 to 25 years. Another study^23^ based on nationally representative data from the US reported significant increases in quit attempts and significant decreases in the average number of cigarettes smoked, even among those with psychological distress, from 1997 to 2015. These European^5^ and US^23^ results are consistent with those of a previous qualitative review,^6^ which found that conversion from current to former smoking, number of quit attempts, and success on a given quit attempt increased rather than declined with time.^6^ Overall, direct evidence provided by our study and indirect evidence from other studies do not support the hardening hypothesis for the general adult population with cigarette use.

We found that nicotine dependence prevalence decreased from 59.52% in 2006 to 56.00% in 2019 (difference, 3.52 percentage points; percentage change, 5.91%) overall among adults who smoked cigarettes. These findings of declines in nicotine dependence among US adults with cigarette use are consistent with the benefits and safety of pharmacotherapy (eg, varenicline) and the benefits of nicotine replacement therapy and counseling that have been reported, even for people with high nicotine dependence.^1,24^ Furthermore, our results suggest that decreases in nicotine dependence prevalence started in 2012, a timeline that contrasts that of a recent study^3^ suggesting that decline in cigarette use may be associated with Affordable Care Act–related increases in health insurance coverage that began in 2014.

Although the recent Surgeon General report on smoking cessation has emphasized that “it is never too late to quit smoking,”^1^ there has been a lack of attention specific to those 50 years and older. Contrary to earlier findings that adults 50 years and older who smoked had lower nicotine dependence prevalence compared with their younger counterparts,^7,8,10,11^ our results revealed that among US adults with cigarette use, those 50 years and older had the highest nicotine dependence prevalence compared with all younger groups. Moreover, nicotine dependence prevalence was positively associated with older age for both men and women. We did not find an interaction effect between age and sex on nicotine dependence. We did find that the pattern of nicotine dependence, which is associated with psychiatric comorbidity,^13^ varied according to age. For example, nicotine dependence prevalence was even higher among those 50 years and older with neither MDE nor SUD than those aged 18 to 34 years with co-occurring MDE and SUD.

High nicotine dependence is associated with increased difficulties in quitting smoking, low quality of life, low work productivity, high health care costs, and high morbidity, disability, and mortality, especially in those 50 years and older, among whom age-related common chronic conditions are often exacerbated by smoking.^1,25,26,27,28^ Smoking can reduce the benefits of medications prescribed for conditions common in later life.^1^ In contrast, smoking cessation in this older population has been found to reduce the increased risk of death and improve recovery from acute and chronic illness.^1^ One systematic review^28^ based on 29 randomized clinical trials reported that for adults 50 years and older, consistent with current clinical practice guidelines, multimodal interventions produced the highest abstinence rates, and pharmacotherapy and behavioral interventions were 2 complementary modalities that improved smoking cessation synergistically. Yet, most older adults who smoked did not try to quit smoking in the past year.^29^ Our results revealed that nicotine dependence prevalence decreased by an AAPC of only 0.3% among those 50 years and older from 2006 to 2019. We also found that differences in nicotine dependence prevalence between those with co-occurring MDE and SUD and those without both conditions were more than 2 times larger for those 50 years and older (18.69 percentage points) than those aged 18 to 49 years (ranging from 7.67 percentage points for ages 18-25 years to 8.28 percentage points for ages 35-49 years).

We also found that nicotine dependence varied by both age and presence of MDE and SUD, with significantly higher nicotine dependence for those with co-occurring MDE and SUD in most age groups and for those with MDE but without SUD in the age groups of 26 to 34 years and 35 to 49 years. High prevalence of depression among adults with cigarette use may be associated with reductions in dopamine receptors (directly or through mechanisms involving the habenula).^30^ Thus, adults with depression or SUD should be prioritized for tobacco control interventions, especially those aged 26 to 49 years with depression.

Notably, primary care clinicians and mental health care professionals can play important roles in encouraging and assisting with smoking cessation efforts among these populations, who may visit them regularly for medical or behavioral health conditions other than nicotine dependence. The recent Surgeon General report^1^ on smoking cessation has covered adults, young adults, and youths, but evidence-based strategies are needed to improve smoking cessation efforts for those 50 years and older and especially those with psychiatric conditions.

This study provided detailed results on how associations of sociodemographic characteristics with nicotine dependence vary by age, highlighting the need to implement evidence-based age-specific tobacco cessation strategies. For example, although previous research^1^ found that low educational attainment was associated with nicotine dependence, our study revealed that differences in nicotine dependence prevalence between those without a high school diploma and those with a college degree were significantly higher among those aged 18 to 34 years than those 50 years and older. Because low educational attainment is associated with lower cessation success,^31^ our results suggest that further research is needed to help understand whether education-related differences in cessation success are markedly higher for young adults than older adults and how to implement evidence-based age-specific nicotine cessation strategies accordingly.

### Limitations

This study has several limitations. First, the cross-sectional nature of NSDUH data precludes the establishment of causal relationships. Second, this study may underestimate the prevalence of nicotine dependence because the NSDUH excluded people experiencing homelessness who were not living in shelters and people who were institutionalized (eg, jail and prison populations), who often have higher nicotine dependence than the general population. Third, this study cannot examine e-cigarette use because the 2006 to 2019 NSDUH did not assess it. Fourth, the NSDUH is a self-report survey and is subject to recall and social desirability bias. Fifth, age is a categorical variable in the NSDUH public use files; thus, this study cannot examine the association of cohort with patterns in nicotine dependence among adults with cigarette use. Future research is needed to examine the associations of age, cohort, and period with nicotine dependence. Sixth, success of cessation treatments, increases in cigarette prices through taxation, and implementation of smoking-free air laws help reduce cigarette consumption and increase cessation rates over time.^1,24,32^ Future research is needed to fully understand how these factors impact patterns in nicotine dependence among US adults with cigarette use. Seventh, it is important to understand patterns in nicotine dependence overall and by age among US adults with cigarette use during the COVID-19 pandemic. However, because the NSDUH data collection modes changed during this period, it is inappropriate to examine any changes between the COVID-19 era (eg, 2020-2022) and the 2006 to 2019 period based on NSDUH data. More research is needed to continue monitoring the patterns.

## Conclusions

This cross-sectional study found that nicotine dependence prevalence declined slightly from 2006 to 2019 among the general adult population with cigarette use and all subgroups 26 years and older. For adults overall with cigarette use, nicotine dependence prevalence had an AAPC decrease of 0.4% from 2006 to 2019. This study also found that both men and women 50 years and older (especially those with MDE and/or SUD) had the highest nicotine dependence prevalence compared with other age groups, highlighting the importance of assisting smoking cessation efforts and addressing nicotine dependence for this older population. Moreover, those aged 18 to 49 years with MDE or SUD also had higher nicotine dependence prevalence than those in the same age group without corresponding psychiatric comorbidities. These results suggest the need to implement evidence-based tobacco cessation strategies that are specific to age and psychiatric comorbidities.

## Notes

Supplement.
Data Sharing Statement
