# Supplementary material for: Patterns and Characteristics of Nicotine Dependence Among Adults With Cigarette Use in the US, 2006-2019
Source: JAMA Netw Open. 2023 Jun 23;6(6):e2319602. doi: 10.1001/jamanetworkopen.2023.19602 (PMC10290248; doi:10.1001/jamanetworkopen.2023.19602)
Supplement: Supplement. — Data Sharing Statement [file jamanetwopen-e2319602-s001.pdf]

Data Sharing Statement

Han. Patterns and Characteristics of Nicotine Dependence Among Adults With Cigarette Use in the US, 2006-2019. *JAMA Netw Open*. Published June 23, 2023. doi:10.1001/jamanetworkopen.2023.19602

Data
Data available: Yes
Data types: Deidentified participant data
How to access data: United States Department of Health and Human Services. National Institutes of Health. National Institute on Drug Abuse, and United States Department of Health and Human Services. Food and Drug Administration. Center for Tobacco Products. Population Assessment of Tobacco and Health (PATH) Study [United States] Restricted-Use Files. Inter-university Consortium for Political and Social Research [distributor], 2021-12-01. Available at: https://doi.org/10.3886/Series606.
When available: With publication

Supporting Documents
Document types: None

Additional Information
Who can access the data: researchers whose proposed use of the data has been approved. A less detailed data set is available to anyone who would like to examine the study data.
Types of analyses: For any purpose
Mechanisms of data availability: with a signed data access agreement as administered by the Inter-university Consortium for Political and Social Research
